# A multicentre cross‐sectional observational study of cancer multidisciplinary teams: Analysis of team decision making

**DOI:** 10.1002/cam4.3366

**Published:** 2020-08-13

**Authors:** Tayana Soukup, Benjamin W. Lamb, Abigail Morbi, Nisha J. Shah, Anish Bali, Viren Asher, Tasha Gandamihardja, Pasquale Giordano, Ara Darzi, James SA Green, Nick Sevdalis

**Affiliations:** ^1^ Centre for Implementation Science King’s College London UK; ^2^ Cambridge University Hospital NHS Trust London UK; ^3^ Department of Surgery and Cancer Imperial College London London UK; ^4^ HeLEX Centre University of Oxford Oxford UK; ^5^ Royal Derby Hospital Derby UK; ^6^ Chelmsford Breast Unit Broomfield Hospital Chelmsford UK; ^7^ Whipps Cross University Hospital Barts Health NHS Trust London UK

**Keywords:** cancer, cancer multidisciplinary team meetings, decision making, multidisciplinary teams

## Abstract

**Background:**

Multidisciplinary teams (MDT) formulate expert informed treatment recommendations for people with cancer. We set out to examine how the factors proposed by the functional perspective of group decision making (DM), that is, interaction process, internal factors (factors emanating from within the group such as group size), external circumstances (factors coming from the outside of the team), and case‐complexity affect the quality of MDT decision making.

**Methods:**

This was a cross‐sectional observational study. Three cancer MDTs were recruited with 44 members overall and 30 of their weekly meetings filmed. Validated observational instruments were used to measure quality of DM, interactions, and complexity of 822 case discussions.

**Results:**

The full regression model with the variables proposed by the functional perspective was significant, *R* ^2^ = 0.52, *F*(20, 801) = 43.47, *P *< .001, adjusted *R* ^2^ = 0.51. Positive predictors of DM quality were asking questions (*P* = .001), providing answers (*P* = .001), team size (*P* = .007), gender balance (*P* = .003), and clinical complexity (*P* = .001), while negative socioemotional reactions (*P* = .007), gender imbalance (*P* = .003), logistical issues (*P* = .001), time‐workload pressures (*P* = .002), and time spent in the meeting (*P* = .001) were negative predictors. Second half of the meetings also saw significant decrease in the DM quality (*P* = .001), interactions (*P* = .001), group size (*P* = .003), and clinical complexity (*P* = .001), and an increase in negative socioemotional reactions (*P* = .001) and time‐workload pressures (*P* = .001).

**Discussion:**

To the best of our knowledge, this is the first study to attempt to assess the factors proposed by the functional perspective in cancer MDTs. One novel finding is the effect of sociocognitive factors on team DM quality, while another is the cognitive‐catch 22 effect: while the case discussions are significantly simpler in the second half of the meeting, there is significantly less time left to discuss the remaining cases, further adding to the cognitive taxation in teams who are now rapidly attempting to close their time‐workload gap. Implications are discussed in relation to quality and safety.

## Graphical abstract

To the best of our knowledge, this is the first study to attempt to assess the factors proposed by the functional perspective in the context of cancer MDT meetings. Validated observational instruments were used to measure quality of decision making, interactions, and complexity of 822 case discussions across 3 cancer teams and 30 of their weekly meetings. One novel finding is unraveling the sociocognitive predictors of team DM quality, while another is the cognitive‐catch 22 effect

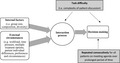

## 1 BACKGROUND

A multidisciplinary approach is accepted as the gold‐standard means of addressing the complex needs of patients with cancer.^1^, ^2^, ^3^, ^4^, ^5^ In the UK, such care planning is routinely (and mandatorily) carried out by a multidisciplinary team (MDT), generally consisting of histopathologists, radiologists, surgeons, cancer nurse specialists (CNSs), and oncologists, in typically weekly or fortnightly meetings (or, tumour boards). There, patients are reviewed (their medical history, imaging, histopathology results, comorbidities, and psychosocial aspects and views on treatments), the treatment options discussed between core disciplines, and treatment recommendations agreed upon. This process is conducted in a sequential manner, usually for several hours at a time, until all patients put forward for MDT review have been discussed.^1^, ^2^, ^3^, ^4^, ^5^

While the MDT approach to cancer care is endorsed widely,^1^, ^2^ evidence of its effectiveness remains unclear.^6^, ^7^, ^8^, ^9^, ^10^, ^11^, ^12^, ^13^, ^14^, ^15^ The pattern of decision making (DM) generally observed in MDT meetings is that of unequal participation in discussion and suboptimal sharing of information, which directly affects the ability of the team to reach a treatment recommendation, as well as whether such recommendations can be subsequently implemented.^6^, ^7^, ^8^, ^9^, ^10^, ^11^, ^12^, ^13^, ^14^, ^15^ MDTs are also affected by the changing economic and political landscape surrounding health care, that is, increasing financial pressures,^16^, ^17^ the rise in cancer incidence,^16^, ^18^ time pressures, severe staff shortages,^19^ and steadily increasing workload MDT’s are under, especially for large teaching hospitals, leading to a rise in frequency/duration of their meetings (with some meetings lasting up to 5 hours).^20^

As cancer MDTs are trying to maximize productivity in the face of increasing workloads and pressures, safety concerns have been raised in the context of MDT meetings with one‐member reporting “*Sometimes we discuss up to 70 patients. This is after a whole day of clinics and we don't finish until gone 19.00. Would you want to be number 70?*”.^4^ Corroborative evidence points to decrements in clinical performance on such sequential tasks.^21^ For example, studies have shown positive association between the quality of DM and cases that are near the start of the MDT meeting,^10^, ^22^ while in other clinical settings, it was shown that the quality of endoscopy performance^23^ and clinical handovers^24^ declines with successive procedures. Indeed, the inherent limitations of human cognition, memory, and attention, effects of fatigue and stress, and the risks associated with distractions can lead to mistakes even with experienced professionals.^25^, ^26^, ^27^, ^28^, ^29^, ^30^, ^31^, ^32^

It is important to consider, therefore, that teams make decisions that are systematically different to lone individuals.^33^, ^34^, ^35^, ^36^, ^37^ Interaction is an advantage in group DM, reducing the overconfidence bias by 24%,^38^ as well as the error rate^39^, ^40^ in comparison to lone DM. This advantage arises during group DM because the information is not only processed cognitively on an individual level but also interactively with other team members.^33^, ^34^, ^35^, ^36^, ^37^ To maintain and increase the DM quality and achieve high level of task efficiency, effective interaction process and communication is critical to help the team move across the different stages of group DM—from problem identification, information sharing, and critical evaluation (of the information and consequences of different options) to formulating the decision and implementing it. Each step has its unique purpose in enabling the group to interact in order to achieve their goal.^33^, ^34^, ^35^

**FIGURE 1:**
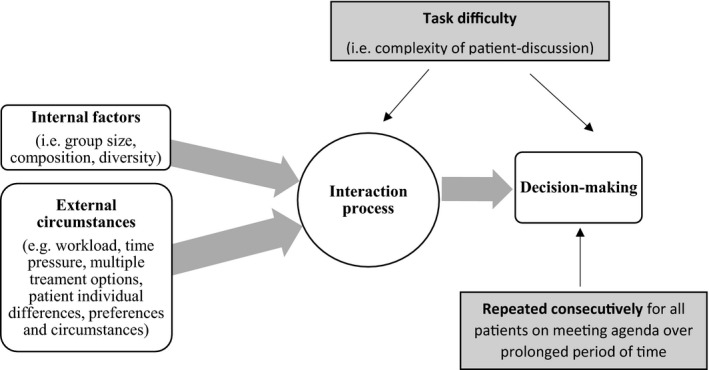
Graphical representation of the functional perspective of group decision making as applied to cancer multidisciplinary team meetings. Note. Reprinted with permission from Soukup, 2017.^52^

From the perspective of patient safety^32^, ^41^, ^42^, ^43^, ^44^, ^45^, ^46^, ^47^, ^48^ as well as the evidence base on group DM,^33^, ^34^, ^35^, ^36^, ^37^ effective interaction process and communication are at the centre of effective team work. The most influential theory of group DM, namely, the *functional perspective*, and the associated research evidence, suggests that variability in performance is attributable to human factors.^32^, ^41^, ^42^, ^43^, ^44^, ^45^, ^46^, ^47^, ^48^ Specifically, the internal factors that come from within the group itself (eg, group size, member composition, gender) as well as the external circumstances (eg, time pressure and workload). These factors impact on group outcomes, such as DM, rendering them more or less effective. Such impacts are moderated by the nature of the task that the group set out to accomplish, that is, its difficulty or complexity, and can be regulated to improve variability and achieve better outcomes.^33^, ^34^, ^35^ Figure 1 demonstrates graphically this hypothesis, which has yet to be explored within the context of MDT meetings and is what we will attempt within the current study.

### 1.1 Study aims and objectives

The aim of the study was therefore to test, for the first time, the functional perspective of group DM in cancer MDTs, operationalized as two specific hypotheses: **(H1)** the interaction process, the internal factors, external circumstances, and case complexity will impact on the quality of DM for patients; and **(H2)** there will be a difference in the interaction process, internal and external factors, and quality of DM between the first and second half of a MDT meeting.

## 2 METHODS

To ensure reporting rigour in our study, we followed the STROBE checklist (Additional File).

### 2.1 Study design

This was a cross‐sectional observational study.

### 2.2 Study setting

The study took place across 3 university hospitals in the Greater London and Derbyshire areas in the UK between September 2015 and July 2016. Three cancer MDTs took part, including breast, colorectal, and gynaecological. Their meetings were video recorded for a period of 3 months.

### 2.3 Participants and sample size

Participants were 44 MDT members: breast MDT = 15, colorectal MDT = 15, and gynaecological MDT = 14. The MDTs had the same composition: surgeons (n = 12), oncologists (n = 6), CNSs (n = 12), radiologists (n = 6), histopathologists (n = 5), and coordinators (who play an administrative role; n = 3). Disciplinary groups were at consultant level during the study period with on average 9 years of experience (min = 2, max = 22). Detailed breakdown of team composition has been published previously.^49^

All cases on the agenda for discussion were video recorded; these included suspected or confirmed cancer, and in breast and gynaecological cancer teams also benign cases. In total, the MDTs discussed 822 patients across 30 MDT meetings during the study. Sample size in terms of the number of MDT meetings observed per team (n = 10) was determined based on prior studies.^10^, ^11^, ^12^, ^13^ Sample size in terms of the observed cases (N = 822) exceeded the minimum needed to detect significance which for F‐tests would be 114 observational units (calculated using G*Power 3 for a priori power analysis with *d* = 0.80, *f* = 0.50; *α* = 0.001, adjusted for power; 1−*β* = 0.95).

Availability sampling was used to identify the teams with a criterion for the study being a cancer MDT from the UK National Health Service (NHS) that represents the commonest types of cancer.

### 2.4 Instruments and variables

Quantitative observational assessments were conducted for each of the 822 case discussions using 3 validated observational instruments. All assessments were conducted from video recordings. What follows is a description of the instruments and variables used for each case discussion, while the copies of the tools can be found in the Additional File.

**DM process** was assessed using the *Metric for Observation of Decision‐making*, (MODe).^9^ MODe has been used previously to assess various cancer MDT meetings and has shown good validity and reliability.^9^, ^10^, ^11^, ^12^, ^22^ It captures the following aspects:

1. **Quality of presented patient information**, which includes 6 variables scored on a behaviourally anchored 5‐point scale, namely, patients’ case history, radiological images, histopathology, psychosocial issues, comorbidities, and their views on treatment options. The sum of the scores for all 6 variables represents overall quality of presented information for a patient with the higher scores indicating better quality.
2. **Quality of contribution to case reviews,** which includes 6 variables scored on a behaviourally anchored 5‐point scale, representing contributions made by the surgeons, oncologists, radiologists, histopathologists, cancer nurse specialist, and the chairperson of the meeting. The sum of the scores for all 6 variables represents overall quality of contribution for a patient with the higher scores indicating better quality.

**Interaction process** between team members was assessed using *Bales Interaction Process Analysis* (Bales IPA).^50^, ^51^, ^52^ This is an observational coding system developed initially with small health‐care teams engaged in weekly diagnostic meetings at Harvard Psychological clinic, and further refined in simulated team meetings. It is based on a principle that a small group represents individuals (2 to 20 people) engaged in a face‐to‐face interaction (in a meeting or series of such meetings) where basic formal similarities irrespective of the context and inherent values exist, that is, “*certain types of action tend to have certain types of effects on subsequent action*”.^50^ As such, it is particularly suitable for cancer MDT meetings: while it was developed and validated within a very similar setting, that is, within weekly health‐care team meetings, it can be used in groups that are diverse in composition, character, and purpose (eg, diagnostic and policy‐forming committees, boards and panels, group therapy and training, work groups, doctor‐patient dyads).^50^, ^51^ For every patient discussed in the meeting, four aspects of MDT interaction were captured using frequency counts by marking the originator and target of each interaction while following the specific rules and framework^50^, ^51^, ^52^; this is as follows:

Positive reactions (socioemotional area)

1. Shows solidarity, cooperation, gives help, raises others status, friendly;
2. Tension release, jokes, laughs, shows satisfaction;
3. Agrees, shows passive acceptance, understands, complies, concurs;

Giving answers (task‐directed area)

- Gives suggestion, direction, instruction, solution, way to achieve goal;
- Gives opinion, evaluation, interpretation, decision making, reasoning;
- Gives orientation, information, repeats, confirms, clarifies;

Asking questions (task‐directed area)

- Asks for orientation, information, repetition, confirmation, clarification;
- Asks for opinion, evaluation, interpretation, decision making, reasoning;
- Asks for suggestion, direction, instruction, solution, way to achieve goal;

Negative reactions (socioemotional area)

- Disagrees, shows passive rejection, unacknowledging (eg, doing something other than the task such as whispering), hesitant, critical, withholds help;
- Shows tension, fear of provoking opposition, frustrated, concerned, asks for help;
- Shows antagonism, deflates other's status, asserts self, autocratic.

**Complexity of each patient** discussed in the meeting was assessed using a psychometrically valid and reliable tool, namely, *Measure of case‐Discussion Complexity* (MeDiC).^52^, ^53^ MeDiC has been developed following a multiphase research process over 18 months with input from cancer specialists throughout at national level in the UK. It demonstrated evidence of reliability and validity in its scores, as well as feasibility in utilisation by both medically and nonmedically trained assessors.^52^, ^53^ MeDiC captures clinical complexity (including pathology, patient factors, and treatment factors) and logistical complexity (administrative and process of care issues) for each patient discussed in the MDT meeting—the former is scored using a checklist principle (with added weight for certain items), while the latter is scored as frequency (tally for every occurrence).

**Internal factors** emanating from within the group were measured as following variables:

1. **Group size** as an overall number of people present at any one case discussion;
2. **Disciplinary composition** as a counter that increases for each additional discipline present during any one case discussion;
3. **Disciplinary distribution** as a categorical variable denoting whether equal number of people within each discipline were present for any one case discussion (0 = unequal disciplinary distribution, 1 = equal disciplinary distribution); and
4. **Gender balance** as 3 separate categorical variables denoting (a) more males, (b) more females, and (c) equal number of males and females present for any one discussion.

**External circumstances** coming from outside the team were measured as following proxies:

1. **Time and workload pressure** as a time‐workload ratio, calculated as the actual/exact time left to discuss the patients from the MDT list (using a video editing software for better precision) divided by the number of patients left to be discussed; higher scores indicate less time‐workload pressure, while the lower values denote increased time‐workload pressure.
2. **Time on task** was captured as (a) *a serial/ordinal counter* that increases for each decision made in the meeting denoting an act of making repeated decisions (ie, a decision count), and (b) a *categorical variable* denoting two equal temporal halves of meetings (ie, the first and second half of the meeting, or temporal meeting halves).

### 2.5 Assessor training and reliability

Training in the use of the 3 observational tools was undertaken by all evaluators prior to the formal scoring during the study. Training is essential to be able to use such tools, and it is a general principle for instruments assessing human factors in clinical environments.^54^ Training was delivered by our team and it involved: (a) explanation of the domains, scales, and their anchors; (b) background reading of peer‐reviewed literature on the tool; and (c) calibration of scoring against an expert evaluator (TS) via scoring a set of MDT videos. Proficiency in scoring was set as an achievement of interassessor reliability of 0.70 or higher between the trainee and expert assessor^54^ across all 3 observational instruments; this was met. Second assessors rated 15%‐20% of case discussions for each tool, respectively, and their scores were calibrated against the main assessor (TS). For Bales’ IPA, scores were calibrated with a social scientist (NJS); for MODe with an academic consultant surgeon (BWL), and for MeDiC with an academic physician (AM). Each evaluator was blind to the other evaluators’ observations.

### 2.6 Bias

*Observer bias* was addressed and reliability of evaluations on the 3 instruments was ensured by having a subset of cases scored by the evaluators in pairs (TS and NS for Bales’ IPA; TS and AB for MeDiC; and TS and BWL for MODe) who were all trained in the use of the instruments. During data collection, each evaluator was blind to the other evaluators’ observations. To reduce the *Hawthorne effect*, that is, teams changing their usual behaviour due to being observed, we adopted a long‐term approach by filming each team for a prolonged period of time, that is, 3 months/12 consecutive weeks, and we excluded the first two meetings in each team from the analysis as they were designed to allow the members to get used to the camera and induce habituation. We also ensured that filming was done discretely by addressing any factors that could serve as a constant reminder to the team that they are being filmed thus allowing the members to ‘forget’ about the camera and continue their working practices as usual. We did this by using a small recording camera, namely, Go Pro, with sound settings and recording light switched off, and using a remote control to start and stop recording. The camera was positioned in the area where it blended in with the background equipment and cables and was out of immediate view of the team.

### 2.7 Statistical analyses

Intraclass correlation coefficient (ICC) analysis was used to assess reliability of evaluations between assessors for each tool. A single measure ICC with the two‐way mixed effects model and an absolute agreement definition was used. ICCs can range between 0 and 1, with higher values indicating better agreement.

**To address H1,** multiple hierarchical linear regression was used with the MODe as the outcome/predicted variable. Variables known to affect DM were entered into the model first; these were internal and external factors and case complexity. New variables of interest, that is, two equal temporal halves, decision count, and interaction process were entered into the model next. The interaction terms between the two equal temporal halves and the interaction process variables were entered last. A detailed report of analyses can be found in the Additional File.

**To address H2,** multivariate analysis of covariance (MANCOVA) was used to gauge differences between the first and second half of meetings on DM, interactions, case complexity, internal factors, and external circumstances. Partial correlation analysis with tumour type as a control variable was conducted between the decision counter and the quality of DM, interactions, case‐complexity, internal factors, and external circumstances. For the categorical items, point‐biserial correlations were run with bootstrapping and tumour type as a stratified variable. See the Additional File.

All analyses were carried out using SPSS^®^ version 20.0 on a dataset available on Zenodo.^55^

## 3 RESULTS

### 3.1 Descriptive statistics

Table 1 provides an overview of the MDT meeting characteristics. The gynaecological MDT had the highest workload and longest meetings, while the colorectal team had the least number of cases for MDT discussion and shortest meeting duration. The colorectal team also spent most time discussing each patient, followed closely by the gynaecological and breast teams. In terms of team composition, breast and colorectal teams had similar number of members attending the meetings; the gynaecological team was the smallest. There were more female members in attendance in breast and colorectal teams, while in the gynaecological team there were more male attendees.

**TABLE 1:** Meeting characteristics of breast, colorectal, and gynaecological cancer team meetings

Item	Breast team			Colorectal team			Gynaecological team			Overall		
	*M*	SD	Min, Max	*M*	SD	Min, Max	*M*	SD	Min, Max	*M*	SD	Min, Max
Meetings observed	10	—	—	10	—	—	10	—	—	30	—	—
Case discussions observed	241	—	—	185	—	—	396	—	—	822	—	—
Case discussions in 1st half of meeting	108	—	—	92	—	—	202	—	—	402	—	—
Case discussions in 2nd half of meeting	133	—	—	93	—	—	194	—	—	420	—	—
Case discussions per meeting	26	3.37	20, 30	20	3.72	15, 27	43	4.94	35, 51	33	11.22	15, 51
Case discussions per meeting in 1st half	6.10	3.47	1, 15	5.09	2.83	1, 12	11.03	6.36	1, 26	8.35	5.72	1, 26
Case discussions per meeting in 2nd half	7.39	4.21	1, 17	5.73	3.31	1, 15	10.57	6.24	1, 26	8.50	5.48	1, 26
Meeting duration (hours and minutes)	01:06	00:12	00:52, 01:31	01:00	00:15	00:40, 01:30	02:52	00:35	01:57, 04:00	01:55	01:00	00:40, 04:00
Time per patient (minutes and seconds)	02:25	01:56	00:06, 10:19	03:02	02:20	00:12, 14:02	02:30	01:57	00:06, 15:25	01:34	02:04	00:06, 15:23
Core MDT members present	11	2.02	5, 15	11	1.76	5, 15	7	1.28	4, 10	9	2.63	4, 15
Females^a^ (%)	63.6	—	—	57.1	—	—	33.3	—	—	51.9	—	—
Males^b^ (%)	36.4	—	—	42.9	—	—	66.7	—	—	48.1	—	—

Note

Reprinted with permission from Soukup, 2017.^52^

Abbreviations: M, mean; MDT, multidisciplinary team; SD, standard deviation.

^a^ Females (n = 27): 3 Surgeons, 4 Oncologists, 2 Pathologists, 11 Cancer Nurse Specialists, 4 Radiologists, and 3 MDT Coordinators.

^b^ Males (n = 17): 9 Surgeons, 3 Radiologists, 2 Oncologists, 2 Pathologists, and 1 Cancer Nurse Specialist.

Table 2 shows descriptive statistics for the composite score of each measure used in the study, that is, the MODe, Bales’ IPA, and MeDiC. It can be seen that colorectal team had the highest mean scores on all 3 measures indicating the highest quality of DM, most intensified interaction process, and most complex case discussions. Breast team closely followed with the scores on the interaction process, however, both breast and gynaecological teams had similar mean scores on the DM quality and case complexity. For a breakdown of descriptives for individual variables within each of the 3 tools across the teams, see the Additional File.

**TABLE 2:** Descriptive statistics for the composite scores of the MeDiC, MODe, and Bales’ IPA

Observational Assessment		Breast team (n = 241)			Colorectal team (n = 185)			Gynaecological team (n = 396)			Overall (N = 822)		
Instrument (score range)	Measuring	*M* (SD)	*Mdn* (*IQR*)	Min, Max	M (SD)	*Mdn* (*IQR*)	Min, Max	*M* (SD)	*Mdn* (*IQR*)	Min, Max	*M* (SD)	*Mdn* (*IQR*)	Min, Max
MeDiC (0 to infinity^c^)	Discussion complexity	3.7 (3.6)	3 (4)	0, 18	6.2 (3.8)	6 (5)	0, 19	3.4 (3.6)	2 (3)	0, 26	4.1 (3.8)	3 (5)	0, 26
MODe (11 to 55^a^)	Decision making	23.3 (6.6)	23 (10)	11, 44	25.6 (5.9)	26 (7)	11, 42	23.2 (5.6)	23 (8)	11, 42	23.8 (6.0)	23 (9)	11, 44
Bales’ IPA (0 to infinity^b^)	Team interactions	28.6 (20.8)	23 (28)	4, 99	29.1 (18.3)	25 (21.5)	4, 96	23.1 (15.1)	19 (18)	4, 99	26.1 (17.9)	21.50	4, 99

Note

Reprinted with permission from Soukup, 2017.^52^

Abbreviations: Bales’ IPA, Bales Interaction Process Analysis; *IQR*, Interquartile Range; *M*, Mean; *Mdn*, Median; MeDiC, Measure of Discussion Complexity; MODe, Metric for Observation of Decision‐making SD, Standard Deviation.

^a^ Composite MODe score is a sum of 11 individual variables each scored on a range of 1 to 5 with higher scores indicating better quality.

^b^ Composite Bales’ IPA score is a sum of 12 variables each scored as a frequency count with higher scores indicating more interactions.

^c^ Composite Complexity score is a sum of 26 (binary) clinical variables and the frequency counts of logistical issues with higher scores indicating more complex case discussions.

### 3.2 Reliability of evaluations

Interassessor agreement was examined using ICCs on a subset of the observed cases: 17% (N = 136) for MeDiC; 20% (N = 158) for MODe; and 15% (N = 117) for Bales’ IPA. For the composite values across the tools, reliability was as follows: ICC = 0.995 (95% CI = 0.994‐0.997) for MeDiC; ICC = 0.934 (95% CI=0.909‐0.952) for MODe; and ICC = 0.993 (95% CI = 0.989‐0.996) for Bales’ IPA tool. For the reliability coefficients for the individual items of MODe and Bales’ IPA, see the Additional File, and for the MeDiC tool, the coefficients have been published previously.^52^, ^53^

**H1: Interaction process, internal and external factors will impact quality of team DM (regression)**

A hierarchical multiple linear regression was run to assess how predictor variables denoting internal and external factors, interaction (Bales’ IPA), and case‐complexity (MeDiC) impact on the DM quality (MODe: contribution and information quality) as an outcome/predicted variable. Table 3/Table 4 shows details of each regression model, and Table 5 a simplified overview of the effects.

**TABLE 3:** Results from hierarchical regression analyzing factors affecting quality of presented information to the team

Domain	Item	Model 1			Model 2			Model 3		
		*B* (*SE*)	β	*P*	*B* (*SE*)	β	*P*	*B* (*SE*)	β	*P*
	Constant	9.04 (0.89)		.001	9.45 (0.87)		.000	9.67 (0.88)		.001
Internal factors	Group size	**0.16 (0.06)**	**0.16**	**.005**	**0.16 (0.06)**	**0.16**	**.006**	**0.16 (0.06)**	**0.16**	**.007**
	More males (0 = g/balance, 1 = more males)	−**1.07 (0.43)**	−**0.10**	**.012**	−**1.05 (0.42)**	−**0.10**	**.012**	−**1.32 (0.42)**	−**0.12**	**.003**
	More females (0 = g/balance, 1 = more females)	−**0.78 (0.26)**	−**0.12**	**.003**	−**0.60 (0.25)**	−**0.09**	**.018**	−**0.70 (0.25)**	−**0.10**	**.006**
	Gender balance (0 = more males and females, 1 = gender balance)^a^	**0.80 (0.26)**	**0.10**	**.002**	**0.64 (0.25)**	**0.08**	**.012**	**0.74 (0.25)**	**0.09**	**.003**
	Disciplinary diversity	0.21 (0.19)	0.05	.263	0.08 (0.19)	0.01	.680	0.02 (0.18)	0.01	.907
	Equal number of people within each discipline (0 = unequal, 1 = equal)	−1.23 (0.66)	−0.06	.064	−1.07 (0.64)	−0.05	.092	−1.21 (0.63)	−0.06	.055
External factors	Time−workload ratio	**0.34 (0.09)**	**0.14**	**.001**	**0.23 (0.08)**	**0.08**	**.007**	**0.27 (0.09)**	**0.11**	**.002**
Task difficulty	Logistical complexity	−**0.54 (0.12)**	−**0.15**	**.001**	−**0.73 (0.12)**	−**0.21**	**.000**	−**0.71 (0.12)**	−**0.20**	**.001**
	Clinical complexity	**0.25 (0.03)**	**0.35**	**.001**	**0.14 (0.03)**	**0.19**	**.000**	**0.13 (0.03)**	**0.19**	**.001**
Cognitive level	Two equal temporal meeting halves (0 = 1^st^ half of the meeting, 1 = 2^nd^ half)				−**0.57 (0.27)**	−**1.11**	**.036**	−0.34 (0.29)	−0.07	.245
	Serial position of treatment decisions within the meeting (ie, the decision count)				0.01 (0.02)	0.03	.574	0.02 (0.01)	0.06	.278
Social level	Giving Answers				**0.05 (0.01)**	**0.19**	**.000**	**0.07 (0.01)**	**0.27**	**.001**
	Asking Questions				**0.08 (0.02)**	**0.21**	**.000**	**0.09 (0.02)**	**0.22**	**.001**
	Positive Reactions				−0.04 (0.03)	−0.05	.259	−0.04 (0.04)	−0.06	.298
	Negative Reactions				−0.02 (0.03)	−0.03	.428	−**0.14 (0.05)**	−**0.18**	**.007**
Social−cognitive level	Giving Answers × 2^nd^ half of meeting							−**0.05 (0.02)**	−**0.19**	**.006**
	Asking Questions × 2^nd^ half of meeting							0.01 (0.03)	0.02	.740
	Positive Reactions × 2^nd^ half of meeting							0.02 (0.06)	0.02	.774
	Negative Reactions × 2^nd^ half of meeting							**0.19 (0.06)**	**0.22**	**.002**

Note

N = 822 case discussions (15 missing cases). Predicted variable is quality of presented information.

**TABLE 4:** Results from hierarchical regression analyzing factors affecting quality of team members’ contribution to patient reviews

Domain	Item	Model 1			Model 2			Model 3		
		*B* (*SE*)	β	*P*	*B* (*SE*)	β	*P*	*B* (*SE*)	β	*P*
	Constant	4.50 (1.36)		.001	6.37 (1.19)		.000	9.67 (0.88)		.001
Internal factors	Group size	0.09 (0.09)	0.05	.321	0.10 (0.08)	0.06	.207	0.09 (0.08)	0.05	.275
	More males	−1.01 (0.66)	−0.06	.127	−0.69 (0.58)	−0.04	.232	−0.93 (0.57)	−0.05	.105
	More females in attendance	−**0.83 (0.40)**	−**0.08**	**.040**	−0.27 (0.35)	−0.03	.434	−0.40 (0.35)	−0.04	.247
	Professional diversity	**0.76 (0.29)**	**0.12**	**.010**	0.31 (0.25)	0.05	.215	0.29 (0.25)	0.04	.252
	Equal number of people within each discipline (0 = unequal, 1 = equal)	−0.81 (1.02)	−.02	.426	−0.60 (0.87)	−0.02	.489	−0.77 (0.87)	−0.02	.376
External factors	Time‐workload ratio	**0.64 (0.13)**	**0.16**	**.001**	**0.30 (0.12)**	**0.07**	**.010**	**0.25 (0.12)**	**0.06**	**.031**
Task difficulty	Logistical complexity	0.02 (0.18)	0.00	.894	−**0.437 (0.16)**	−**0.07**	**.007**	−**0.44 (0.16)**	−**0.07**	**.006**
	Clinical complexity	**0.52 (0.04)**	**0.44**	**.001**	**0.20 (0.04)**	**0.17**	**.000**	**0.20 (0.04)**	**0.17**	**.001**
Cognitive level	Two equal temporal halves (0 = 1^st^ half of the meeting, 1 = 2^nd^ half)				−0.53 (0.37)	−0.06	.147	−**1.51 (0.52)**	−**0.18**	**.004**
	Serial position of treatment decisions in the meeting (ie, the decision count)				−**0.087 (0.02)**	−**0.22**	**.001**	−**0.07 (0.02)**	**0.24**	**.001**
Social level	Giving Answers				**0.11 (0.01)**	**0.25**	**.000**	**0.10 (0.02)**	**0.27**	**.001**
	Asking Questions				**0.17 (0.02)**	**0.26**	**.000**	**0.13 (0.03)**	**0.20**	**.001**
	Positive Reactions				0.06 (0.04)	0.05	.168	0.08 (0.05)	0.07	.123
	Negative Reactions				0.08 (0.04)	0.06	.056	−0.01 (0.07)	−0.01	.837
Sociocogniitve level	Giving Answers × 2^nd^ half of meeting							0.00 (0.03)	0.01	.936
	Asking Questions × 2^nd^ half of meeting							**0.14 (0.04)**	**0.15**	**.002**
	Positive Reactions × 2^nd^ half of meeting							−0.04 (0.08)	‐0.02	.647
	Negative Reactions × 2^nd^ half of meeting							0.12 (0.08)	0.09	.149

**TABLE 5:** Overview of the sociocognitive predictors significantly impacting the teams’ clinical decision‐making process

Sociocognitive factors	Quality of presented information to the team	Quality of contribution to case discussion by team members
Group size	+	No effect
Gender balance within the team	+	No effect
Clinical complexity of patient	+	+
Asking questions	+	+
Giving answers	+	+
More male attendees	−	No effect
More female attendees	−	−
Time‐workload pressures	−	−
Time spent on task	No effect	−
Logistic complexity	−	−
Negative reactions	−	−

Note

In green are factors with positive effect, and in red those with the negative effect on the team's decision making.

### 3.3 Quality of contribution to case reviews (Table 3)

The full model (Model 3) with all variables and interaction terms included was significant, *R* ^2^ = 0.52, *F*(20, 801) = 43.47, *P *< .001, adjusted *R* ^2^ = 0.51. The model with the encompassing significant variables explained 51% of the variance in the contribution scores. When all variables were held constant, the contribution score increased by 0.25 for each unit increase in time‐workload ratio. For each one score increase in *case complexity*, it increased by 0.20. For each frequency count increase in *giving answers*, it increased by 0.10, and for *asking questions* the increase was 0.13. In contrast, when all variables were held constant, the contribution score decreased by 0.07 with *each decision made* in the meeting, while in the second half of the meeting decrease was at 1.51. For each instance increase in the frequency of logistical issues, the contribution score decreased by 0.44. Logistical issues refer to administrative, process, attendance, and equipment issues experienced by the team during the meeting (the type and frequency of these issues across the teams is in Additional File).

The simple slopes analysis revealed a significant **positive** relationship between *asking questions* and *contribution quality* (*B* = 0.26, *SE* = 0.02) in the second half of the meeting, *P* = .001, and in the first half, the relationship was also significant but weaker (*B* = 0.13, *SE* = 0.03), *P* = .001. The coefficient of the interaction term (*B* = 0.14, *SE* = 0.04) was significant (*P* = .002), indicating that the variable denoting two *equal temporal halves* positively moderated the relationship between *asking questions* and *contribution quality*. The remaining interactions were nonsignificant.

### 3.4 Quality of presented patient Information (Table 4)

The full model (Model 3) with all variables and interaction terms included was significant, *R* ^2^ = 0.28, *F*(20, 801) = 15.35, *P *< .001, adjusted *R* ^2^ = 0.26. The model with the encompassing significant variables explained 26% of the variance in the information score. When all variables were held constant, the information score increased by 0.16 for each one‐person increase in the *group size*. For each one increase in the *time‐workload ratio*, it increased by 0.27, and for one‐score increase in the case *complexity*, the increase was by 0.13. Information score also increased by 0.27 with one‐instance increase in the frequency of *giving answers*, and by 0.09 with *asking questions*. In contrast, when there were *more males* in the team, the information score decreased by 1.32, while when there were *more females*, it decreased by 0.68. With one‐instance increase in the frequency of *logistical issues* in the meeting, the information score decreased by 0.71. And for each instance of *negative reactions* between the members, the information score decreased by 0.14.

The simple slopes analysis revealed a significant **positive** relationship between g*iving answers* and *information quality* (*B* = 0.07, *SE* = 0.01) in the second half of meetings, *P* = .001, and in the first half, the relationship was also positive, but nonsignificant (*B* = 0.02, *SE* = 0.01), *P* = .12. The coefficient of the interaction term (*B* = −0.05, *SE* = 0.02) was significant (*P* = .006), indicating that the variable denoting two equal temporal halves positively moderated the relationship between *giving answers* and *information quality* (especially, *gives orientation and information*).

The simple slopes analysis also revealed a significant **negative** relationship between *negative reactions* and *information quality* (*B* = −0.14, *SE* = 0.05) in the second half of the meeting, *P* = .006, and in the first half, the relationship was nonsignificant (*B* = 0.05, *SE* = 0.04), *P* = .166. The coefficient of the interaction term (*B* = −0.19, *SE* = 0.06) was significant (*P* = .002), indicating that the variable denoting two equal temporal halves negatively moderated the relationship between *negative reactions* and *information quality*; that is, relationship between *negative reactions* and *information* quality became significantly negative in the second half (especially, *disagrees, tension, and antagonism*).

### 3.5 H2: Differences between the first and second half of the meeting

**MANCOVA** (Table 6) with Hotelling's T was conducted to gauge differences in two equal temporal meeting halves on the quality of DM, interaction process, case complexity, internal factors, and external circumstances, while controlling for tumour type. There was significant difference between the first and second half of meetings on the combined dependent variables, *F*(11, 809) = 21.56, *P *< .001; Hotelling's Trace = 0.29, partial *η* ^2^ = 0.23. Follow‐up univariate ANOVAs showed that the scores on the *information* and *contribution quality* were significantly lower in the second half, as well as the certain aspects of team interaction process, including *asking questions* and *positive reactions*. In contrast, *negative reactions* were significantly higher in the second half. In terms of the internal and external factors, *time‐workload ratio* and *clinical complexity* were significantly lower in the second half, while *group size* was significantly higher. The remaining variables did not reach significance.

**TABLE 6:** Differences in scores between two equal temporal halves of the meeting

Domain	Item	1st half of the meeting (n = 401)		2nd half of the meeting (n = 421)		One‐way ANOVA		
		M (SD)	*Mdn* (*IQR*)	M (SD)	*Mdn* (*IQR*)	F	*df*	*P*‐value
Decision making (MODe)	Quality of information	12.3 (2.71)	12 (3)	11.59 (2.44)	11 (3)	15.71	1	**.001**
	Quality of contribution	13.10 (3.84)	13 (5)	10.64 (4.37)	9 (6)	74.93	1	**.001**
Interactions (Bales’ IPA)	Giving answers	14.26 (9.91)	12 (11)	13.72 (10.11)	11 (10)	0.72	1	.397
	Asking questions	7.15 (7.53)	5 (8)	5.22 (5.58)	4 (4)	17.67	1	**.001**
	Positive reactions	4.04 (4.05)	3 (4)	2.47 (2.89)	1.5 (4)	50.54	1	**.001** ^a^
	Negative reactions	2.15 (2.87)	1 (3)	3.17 (3.63)	2 (5)	17.91	1	**.001** ^a^
Task difficulty (MeDiC)	Logistical complexity	0.53 (0.71)	0.00 (1)	0.52 (0.75)	0.00 (1)	0.04	1	.848
	Clinical complexity	4.00 (3.68)	3 (5)	3.19 (3.50)	2 (4)	14.03	1	**.001** ^a^
External factors	Time‐workload ratio	2.62 (0.63)	2.61 (0.85)	2.37 (1.32)	2.21 (0.87)	10.37	1	**.001**
Internal factors	Group size	9.02 (2.56)	8 (4)	9.60 (2.67)	10 (5)	9.18	1	**.003**
	Disciplinary diversity	5.41 (0.64)	5 (1)	5.37 (0.66)	5 (1)	5.54	1	.019
		Count	—	Count	—	Fisher's Exact Test		
	Equal number of people within each discipline^b^	10	—	4	—	—	—	.109
	Unequal number of people within each discipline^b^	392	—	416	—	—	—	.109
	More males^b^	29	—	26	—	—	—	.327
	More females^b^	319	—	351	—	—	—	.127
	Gender balance^b^	54	—	43	—	—	—	.161

Note

N = 822 case discussions (15 missing cases). Significance set at 0.005. Significances are in boldface. Reprinted with permission from Soukup, 2017.^52^

Abbreviations: Bales’ IPA, Bales Interaction Process Analysis; *df*, degrees of freedom; IQR, Interquartile Range; *M*, Mean; *Mdn*, Median; MeDiC, Measure of Discussion Complexity; MODe, Metric for the Observation of Decision‐making; SD, Standard Deviation.

^a^ Dichotomous variables.

^b^ There are differences between tumour types with gynae showing the lowest mean for *negative reactions* (1.41) in comparison to colorectal (3.80) and breast (3.90); for *positive reactions,* breast shows the highest mean (4.77) in comparison to colorectal (2.80) and gynae (2.51); for *clinical complexity,* the mean is highest with the colorectal team (5.28), and lowest for breast (3.28) and gynae (2.99).

**Partial correlation** (Table 7) analysis controlling for tumour type was conducted to further explore the differences between the first and second half of meetings; these corroborated MANCOVA results. Negative correlations with the decision counter were evident for the *DM quality*, *interaction process*, *case complexity*, and *disciplinary distribution*. In contrast, positive correlation with the decision counter was evident for the g*roup size* and *professional diversity* in the first half of the meeting, while in the second half, the association was negative.

**TABLE 7:** Correlation coefficients for the relationship with the serial decision count across two equal temporal halves of the meetings and the entire dataset

Domain (tool)	Item	Serial position of treatment decisions within the 1st half of meetings, only (n = 401)	Serial position of treatment decisions within the 2nd half of meetings, only (n = 421)	Serial position of treatment decisions in the meetings across the entire dataset (N = 822)
Decision making (MODe)	Quality of information	0.09	−0.03	−0.10**
	Quality of contribution	−0.11*	−0.29***	−0.36***
	Quality of decision making (composite score)	−0.03	−0.23***	−0.30***
Interactions (Bales’ IPA)	Giving answers	0.09	−0.12*	−0.02
	Asking questions	−0.04	−0.28**	−0.21***
	Positive reactions	−0.03	−0.04	−0.19***
	Negative reactions	−0.08	−0.19**	0.02
	Frequency of interactions (composite score)	0.02	−0.18***	−0.13***
Task difficulty (MeDiC)	Logistical complexity	−0.16**	−0.22**	−0.12***
	Clinical complexity	−0.08	−0.24**	−0.20***
	Composite score	−0.11*	−0.27***	−0.21***
External factors	Time‐workload ratio	−0.04	−0.15**	−0.15***
Internal factors	Group size	0.12*	−0.48**	−0.06
	Disciplinary diversity	0.18**	−0.34**	−0.15***
	Equal number of people within each discipline^***^	−0.15**	−0.00	−0.09*
	More males^***^	−0.16**	−0.26**	−0.17***
	More females^***^	0.10*	0.05	0.09*

Note

Reprinted with permission from Soukup, 2017.^52^

^a^ Categorical variables. *Partial correlations* controlling for tumour type were conducted for the continuous variables and cross‐checked against non‐parametric correlations; no differences to statistical conclusions were found. *Point‐biserial* correlations were conducted for categorical variables^a^; test comparisons for validation were conducted against Mann‐Whitney and no differences to statistical conclusions were found. Bootstrapping method was used throughout with 5000 bootstrap samples, tumour type as a stratified variable, and bias‐corrected confidence estimates to ensure power.

*** *P *< .001,

** *P* < .01,

* *P* < .05.

## 4 DISCUSSION

The aim of the study was to test functional perspective of group DM in cancer MDT meetings for the first time, and explore 2 specific hypotheses.

### 4.1 H1: Interaction process, internal and external factors will impact on the quality of team DM

This hypothesis was supported. We found that the variables proposed by the functional perspective, that is, the interaction process, internal factors, external circumstances, and case complexity explained more than half of the total variance in the contribution, and a quarter in the information quality.

Specifically, the study found that **barriers** to team DM in the meetings were negative socioemotional reactions (ie, antagonism, tension, and unacknowledgment), gender imbalance, time spent discussing the cases, time‐workload pressures, and logistical complexities, that is, the administrative, process, attendance, and equipment issues (type and frequency of these issues are in the Additional File). The biggest inhibitory impact on information sharing during DM was due to more men in attendance, while having more women also had an impact, but to a lesser extent. Also, the inhibitory impact of negative socioemotional reactions on information sharing intensified in the second half of the meeting when, arguably, time‐on‐task effects kicked in with self‐regulation of emotional responses becoming more challenging.^21^, ^56^, ^57^ The biggest impact on MDT members’ contribution to case reviewing was as a result of time spent discussing the high volume of patients. For every treatment recommendation made in the meeting, the contribution by the MDT was reduced with the biggest reduction seen in the second half of the meeting; a finding that corroborates previous research on time‐on‐task effects, documented also in MDT meetings.^10^, ^21^, ^22^

**Facilitators** to team DM were some of the fundamental interactional properties during group tasks, such as asking questions and providing answers to these questions, as well as the size of the team, gender balance during the meeting, and clinical complexity of the cases under discussion since more complex cases require wider disciplinary engagement and increased information coverage, providing further external validation to the MeDiC tool.^52^, ^53^ The facilitative effect of asking questions when reviewing the patients strengthened in the second half of the meeting, indicating a greater need to prompt the team to engage in discussion. That is, as the meeting progressed, the MDTs’ readiness to contribute to discussion was reduced, hence, asking questions served as a prompt to maintaining engagement and focus through seeking of further information and clarification.^58^ The facilitative effect of giving answers to the questions asked also strengthened in the second half, despite an overall decline in information quality. It appears that the team members tended to express their opinions, feelings, and wishes more frequently in the second half, while providing less orientation and factual information, thus, impacting negatively on the information quality. This is in contrast to the first meeting half where the reverse pattern was seen. It is possible that due to time‐on‐task effects, in the second half, the team begun to rely more on the “general feeling” and less on the extensive detail in their DM, corroborating previous research.^21^, ^59^, ^60^, ^61^

### 4.2 H2: Differences between the first and second half of the meeting

This hypothesis was also supported. We found a reduction in the quality of DM in the second half of the meeting with a decline evident with each subsequent treatment recommendation made and the optimal period of DM being for the first 20 patients. A decrease was also evident in some of the identified facilitators, that is, frequency of asking questions and giving answers, and an increase in the barrier, that is, negative socioemotional reactions. Our finding is in line with the literature showing that performance deteriorates over sequential tasks ^10^, ^21^, ^22^ and goes some way to answer the recent call to test this premise on large sample sizes,^62^ which our study exceeds with 822 cases.

Another novel finding is in relation to the clinical complexity of the patients discussed in the MDT meetings. It appears that the complexity is significantly higher in the first half of the meeting across teams, which indicates that the MDTs priorities more complex cases by default (this finding emerged as part of the analysis as there was no explicit allocation of cases for the purposes of the study). This also means that the MDT members engage in more complex DM in the first half when they formulate more difficult, that is, less straightforward, treatment recommendations.

However, while the cases are significantly simpler in the second half of the meeting, the time‐workload pressure is significantly higher, that is, there is less time left to discuss the remaining cases. As the MDTs, therefore, attempt to close the time‐workload gap in order to complete their meeting within the allocated time slot and review all patients put forward for MDT discussion, they also increase the pace with which they formulate decisions. This pressure may inadvertently intensify the cognitive load in team members who had already engaged in a series of complex case discussions in the first half (and possibly other clinical commitments prior to the meeting). For the teams’ cognitive resources, this is arguably a ‘*cognitive‐catch 22’*—that is, although cases are simpler in the second half, there is less time left to discuss them, and the team is more cognitively taxed due to the preceding complex cases. Unstable group composition between the first and second half of the meetings may also add to these effects as the team size and disciplinary diversity begin to decrease in the second half resulting in a smaller and less professionally diverse MDT. This is important to consider because group size and disciplinary diversity were shown to be positively associated with DM^6^ and are reported to be important for cancer MDTs’ ability to reach a recommendation.^8^, ^11^, ^12^

### 4.3 Implications

To minimise the impact of negative socioemotional reactions, we propose that the meeting chair does not contribute clinically.^3^, ^12^ We believe that this will help the chair to effectively navigate interaction and communication process between disciplines, while ensuring a more uniform decision‐making process for all patients reviewed by the MDT. To address the negative impact of gender imbalance, we propose that the process of staff selection for MDT meetings captures the professional diversity that is necessary for optimal clinical decision making, while taking into consideration the gender of team members. While we understand that due to competing clinical commitments and staff shortages, balanced staff selection may be challenging, it is essential that attempts are made in order to ensure improved functioning and service quality. Adequate preparation time ahead of the meeting is also needed to address the logistical issues, that is, administrative errors and process issues that were found to impeded team DM. Preparation for the meeting could be improved by using a checklist,^3^, ^63^ for example, to ensure that all essential information is available for the meeting.

Regarding the negative impact of time‐workload pressures, time on task, and the associated ‘cognitive‐catch 22’, we propose cost‐effective cognitive‐behavioural strategies taking into account the intensity and complexity of the workload during working hours, such as, a maximum limit to the number of cases allowed for a single meeting and a mandatory short break (as practiced in the aviation industry).^21^, ^22^ With the guidance on streamlining MDTs now published in the UK,^64^, ^65^ the mandate for discussing all cancer cases at the MDT meeting no longer exists. This means that only complex patients, requiring true multidisciplinary input, would be discussed, while patients on predetermined clinical guidelines would be registered, but not discussed. Effectively, this approach could help reduce the time all MDT members spend in the meetings,^64^, ^65^ while also, indirectly, preserving optimal DM by counteracting the cognitive‐catch 22 and time‐on‐task effects.

### 4.4 Limitations

Our findings need to be interpreted within certain limitations. First is the Hawthorne effect. In line with the ethical and regulatory approvals of participating NHS organisations in the UK, we sought informed consent from team members which meant that they knew that they were going to be filmed (ie, there was no deception). We have, therefore: (a) adopted a long‐term approach by filming each team for a prolonged period of time, (b) excluded the first two meetings in each team from the analysis, (c) ensured that filming was done discretely, and (d) used validated observational tools scored by trained evaluators in pairs blind to one another's observations. Lastly, while the current study is focused on DM process at the point of the MDT meeting, we have not linked these to clinical, patient‐related outcomes. As a result, the safety and clinical implications of this analysis remain exploratory and are not yet equated to clinical outcomes. Moreover, while the sample size is adequately large (N = 822) for an observational study, it represents the most common cancers within the English NHS. Replication of the study on other cancer, teams, and health‐care systems may be needed to determine generalizability of the findings.

### 4.5 Further research

Further research is needed to address the impact of (a) streamlining of MDTs on the quality of DM, (b) internal and external factors on the team's interaction process, and (c) sociocognitive predictors including time‐on‐task effects on the quality of MDT decisions, which could be assessed not only against clinical guidelines, postmeeting implementation of MDT decision, and treatment compliance but also patient satisfaction with the MDT recommendation. This is important because evidence from cognitive psychology shows that prolonged time spent on sequential tasks leads to multiple cognitive pitfalls, including reduced ability to effectively evaluate choices and all available information, but also to making riskier and more impulsive decisions above and beyond one's experience with the task and personality traits.^23^, ^24^, ^25^, ^26^, ^27^, ^28^, ^29^, ^30^, ^31^, ^32^ For cancer MDTs, this could mean poor (or wasteful) clinical outcomes with implications for safety.

The current study focused on specific variables (group size, diversity, etc) in order to test the functional perspective. These variables were chosen in a feasible/pragmatic manner taking into account the resources available at the time. In addition, they were subject to limitations common in most research that involves health‐care professionals, and which arise from system‐ and organization‐related barriers, such as lack of time and clinical work naturally taking priority over participation in research.^66^, ^67^ Nonetheless, further research should explore how to adequately measure other potential influences that were beyond the scope of the present study, such as distractions, burnout, team climate, perceived workload, and cognitive measures (eg, executive control using Stroop task). Developing valid and reliable methods and tools to accurately capture MDT processes is important in building a comprehensive picture of how best to implement the MDT model of care. As health care is increasingly moving toward MDT model, such knowledge will become critical.

## 5 CONCLUSION

To the best of our knowledge, this is the first study to attempt to assess the factors proposed by the functional perspective. One novel finding is the effect of sociocognitive factors on team DM quality, while another is the cognitive‐catch 22 effect with implications for quality and safety. Our methodological approach could be profitably applied to other cancer MDTs. Additional files

## Ethics approval and consent to participate

The study was granted ethical and regulatory approvals by the North West London Research Ethics Committee (JRCO REF. 157441), and also locally by the R&D departments of the participating NHS Trusts. Informed consent was sought from all participants. The study was adopted by the National Institute for Health Research Clinical Research Network Portfolio.

## CONFLICT OF INTERESTS

BL and TS received funding for training MDTs in assessment and quality improvement methods in the United Kingdom. TS serves as a consultant to F. Hoffmann‐La Roche Diagnostics providing advisory research services in relation to innovations for multidisciplinary teams and their meetings in the United States. NS is the Director of London Safety & Training Solutions Ltd, which provides patient safety and quality improvement training and advisory services on a consultancy basis to hospitals and training programs in the UK and internationally. JG is the Director of Green Cross Medical Ltd that developed MDT FIT for use by National Health Service Cancer Teams in the UK. The other authors have no conflicts of interest to report.

## AUTHOR CONTRIBUTIONS

TS has made substantial contribution to the conception and design of the study (as part of her PhD from Imperial College London). TS, BWL, NS, and JSA have made substantial contribution to the analysis and interpretation of the data. All authors have made substantial contribution to the acquisition of data; drafting the manuscript and revising it critically for important intellectual content; have given final approval of the version to be published; and have agreed to be accountable for all aspects of the work in ensuring that questions related to the accuracy or integrity of any part of the work are appropriately investigated and resolved.

## Supporting information

Supplementary Material

## Data Availability

The anonymized dataset supporting this study is available in Zenodo, a research data repository, https://zenodo.org/record/582272#.XntHvoj7Q2w under the Creative Commons Attribution Non‐Commercial Non‐Derivative 4.0 license. Researchers are free to reuse and redistribute the dataset on the condition that they attribute it, that they do not use it for commercial purposes, and that they do not alter it. For any reuse or redistribution, researchers must make clear to others the license terms of this work, and reference the dataset accordingly (see reference [55] for an example).
