# Supplementary material for: A multicentre cross‐sectional observational study of cancer multidisciplinary teams: Analysis of team decision making
Source: Cancer Med. 2020 Aug 13;9(19):7083–99. doi: 10.1002/cam4.3366 (PMC7541152; doi:10.1002/cam4.3366)
Supplement: Supplementary file 1 — Supplementary Material [file CAM4-9-7083-s001.pdf]

ADDITIONAL FILE related to 'A multicentre cross-sectional observational study of cancer multidisciplinary teams: analysis of team decision-making'

LIST OF CONTENT:

1. Data analysis and further results

1.1 H1: Interaction process, internal and external factors will impact quality of team decision-making (regression)

1.2 H2: Differences between the 1st and 2nd half of the meeting

2. Descriptive statistics across individual items of the tools used in the study

2.1 MeDiC (case-complexity)

2.2 MDT-MODe (decision-making)

2.3 Bales' IPA (interaction process)

3. Interrater reliability across individual items of the tools used in the study

3.1 MeDiC (case-complexity)

3.2 MDT-MODe (decision-making)

3.3 Bales' IPA (interaction process)

4. A list of logistical issues encountered by the teams

4.1 Type of logistical issues

4.2 Frequency of logistical issues

5. Copy of the tools used in the study

5.1 MeDiC (case-complexity)

5.2 MDT-MODe (decision-making)

5.3 Bales' IPA (interaction process)

6. STROBE statement-checklist

1. Data analysis and further results

1.1 H1: Interaction process, internal and external factors will impact quality of team DM (regression)

A hierarchical multiple linear regression was run on the variable denoting the following: two equal temporal halves (1st and 2nd half of the meeting), decision count (i.e. a serial counter that increases for every treatment recommendation made in the meeting), quality of interactions (i.e. positive reactions, giving answers, asking questions, and negative reactions), internal factors (group size, disciplinary composition, disciplinary distribution, gender ratio), external factors (time and workload pressure) and the task-difficulty (clinical and logistical complexity) as predictor variables, and the quality of DM (i.e. information and contribution scores) as an outcome variable. The aim was to assess the following:

a. the relationship between predictor variables and the outcome,

b. if the addition of two equal temporal halves, decision counts, and interactions improved the prediction of the outcome over and above the internal and external factors and patient-discussion complexity,

c. if there is an increase in variation explained by the addition of interaction terms, between two equal temporal halves as a moderator and the quality of interactions, to a main effects model.

There was linearity as assessed by partial regression plots and a plot of studentized residuals against the predicted values. There was independence of residuals, as assessed by a Durbin-Watson statistic of 1.50 for contribution scores, and 1.69 for information scores. There was homoscedasticity, as assessed by visual inspection of a plot of studentized residuals versus unstandardized predicted values. There was no evidence of multicollinearity, as assessed by Tolerance values greater than 0.1 (Hair et al., 2014); this was achieved throughout (the lowest was 0.27). In addition, none of the predictor variables had correlations greater than 0.7 (the highest *r* was 0.56). There were two studentized residuals greater than ±3 standard deviations, no leverage values greater than 0.2, and one deleted value for Cook's distance above 1. The assumption of normality was met, as assessed by Q-Q Plots.

Quality of team contribution to case discussion

The full model of two equal temporal halves, decision counts, quality of interactions, complexity, internal and external factors, and interaction terms (Model 3) was statistically significant, R^2 = .52, *F*(20, 801) = 43.47, *p* < .001, adjusted R^2 = .51. Hence, 51% of the variance in the contribution scores can be explained by the model and the encompassing significant variables. The following items were found to be positive significant predictors of the contribution score. When all other variables are held constant, the contribution score increased by 0.25 for each one unit increase in time-workload ratio. For each one score increase in the *complexity*, it increased by 0.20. For each frequency count increase in *giving answers*, it increased by 0.10, while for *asking questions* the increase is 0.13. In contrast, the following variable were found to be negative predictors of the contribution score. When all other variables are held constant, the contribution score decreased by 0.07 with *each subsequent decision made* in the meeting, while in the 2nd half of the meeting decreased was 1.51. For each instance increase in the frequency of logistical issues, the contribution score decreased by 0.44.

Moreover, the addition of the interaction process variables, two equal temporal meeting halves and decision counts (Model 2) led to a statistically significant increase in R^2 of .18, *F*(6, 805) = 48.92, *p* < .001, indicating an increase of 18% in total variation explained. The addition of the interaction terms between quality of interactions variables and two equal temporal halves (Model 3) also led to a statistically significant increase in R^2 of .01, *F*(4, 801) = 3.86, *p* < .01. Hence, the two equal temporal halves moderated the effect of interactions on the **quality of contribution** to discussion as evidenced by a statistically significant increase in the total variation explained by 1%.

Further, the simple slopes analysis revealed that there was a statistically significant positive relationship between **asking questions** and quality of contribution to discussion (*B* = 0.26, *SE* = 0.02) in the 2nd half of the meeting, *p* = .001, and in the 1st half of the meeting the relationship was also significant but weaker (*B* = 0.13, *SE* = 0.03), *p* = .001. The coefficient of the interaction term (*B* = 0.14, *SE* = 0.04) was statistically significant (*p* = .002) indicating that the variable denoting two equal temporal halves moderated the relationship between asking questions and quality of contribution to discussion – i.e. positive effect of *asking questions* on

the quality of contribution to discussion increased in the 2nd half of the meeting. The remaining interactions were non-significant.

Quality of presented patient Information by the team

The full model of two equal temporal halves, decision counts, quality of interactions, complexity, internal and external factors, and interaction terms (Model 3) was statistically significant, R^2 = .28, *F*(20, 801) = 15.35, *p* < .001, adjusted R^2 = .26. Hence, 26% of the variance in the quality of presented information can be explained by the model and encompassing significant variables. The following items were found to be positive significant predictors of the information score. When all other variables are held constant, the information score increased by 0.16 for each one-person increase in the *group size*. For each one increase in the *time-workload ratio*, it increased by 0.27, and for one score increase in the *complexity*, the increase was by 0.13. Information also increases by 0.27 with one instance increase in the frequency of *giving answers*, and by 0.09 with *asking questions*. The negative predictor variables were as follows. When there are *more males* in the team as opposed to females, the information score decreases by 1.32, while when there are *more females*, it decreases by substantially less i.e. 0.68. With one instance increase in the frequency of *logistical issues* in the meeting, the information score decreases by 0.71. And for each instance of *negative reactions* between the members, the information score decreases by 0.14. Regression coefficients and standard errors can be found in Table 8 (below).

Moreover, the addition of the quality of interactions variables, two equal temporal halves and decision counts (Model 2) led to a statistically significant increase in R^2 of .06, *F*(6, 805) = 10.67, *p* < .001, which is an increase of 5.9% of the total variances explained. The addition of the interaction terms between quality of interactions variables and temporal meeting halves (Model 3) also led to a statistically significant increase R^2 of .02, *F*(4, 801) = 5.21, *p* < .001. Hence, the temporal meeting halves moderated the effect of interactions on the quality of presented information as evidenced by a statistically significant increase in the total variation explained by 1.9%. See Table 9 for full details on each regression model.

More to the point, the simple slopes analysis revealed that there was a statistically significant positive relationship between **giving answers** and quality of presented information (*B* = 0.07, *SE* = 0.01) in the 2nd half of the meeting, *p* = .001, and in the 1st half of the meeting the

relationship was also positive, but non-significant (*B* = 0.02, *SE* = 0.01), *p* = .12. The coefficient of the interaction term (*B* = -0.05, *SE* = 0.02) was statistically significant (*p* = .006) indicating that the variable denoting two equal temporal halves moderated the relationship between giving answers and quality of presented information – i.e. the effect of *giving answers* on the quality of presented patient information increased in the 2nd half of the meeting (particularly *gives orientation*).

In addition, the simple slopes analysis also revealed that there was a statistically significant negative relationship between **negative reactions** and quality of presented information (*B* = -0.14, *SE* = 0.05) in the 2nd half of the meeting, *p* = .006, and in the 1st half of the meeting the relationship was non-significant (*B* = 0.05, *SE* = 0.04), *p* = .166. The coefficient of the interaction term (*B* = -0.19, *SE* = 0.06) was statistically significant (*p* = .002) indicating that the variable denoting two equal temporal halves negatively moderated the relationship between negative reactions and quality of presented information – i.e. relationship between *negative reactions* and quality of presented information became negative and significant in the 2nd half of the meeting (particularly, *disagrees, shows tension and antagonism*).

1.2 H2: Differences between the 1st and 2nd half of the meeting

MANCOVA

MANCOVA with the Hotelling's T was conducted on the dataset[58] to determine differences in two equal temporal halves of the meetings (i.e., the 1st and 2nd half of the meeting) on the quality of DM, interactions, complexity, internal and external factors while controlling for tumour type.

Residual analysis, testing the assumptions of ANOVA, showed that although Shaprio-Wilk test ($p < .05$) was significant for each group combination of the IVs, the Normal Q-Q Plots showed that data is normally distributed (some outliers were observed, however). Sensitivity analysis was also done on the log transformed data (with no outliers) and it was cross-validated against non-parametric tests (Mann-Whitney) - no meaningful changes in statistical conclusions were found. ANOVA is considered robust to any deviations from normality especially with large sample sizes (Maxwell and Delaney, 2004), such is this dataset, *N* = 822.

Boxplot and Mahalanobis distance ($p < 0.001$) showed univariate and multivariate outliers, which upon inspection were found to be valid, genuine data points and not measurement

errors. They also do not materially affect the results, as assessed by re-running the analysis with no outliers (on transformed and non-transformed data). ANOVA is considered robust to outliers if the sample size is large enough, therefore, the outliers were not removed. The assumption of the homogeneity of variance-covariance matrices was violated, as assessed by Box's M test (p = .001). Hence to preserve statistical power, a lower level of significance was accepted, using **Bonferroni adjusted alpha level of .005** throughout (Jaccard, 1998). The remaining assumptions were met - there were linear relationships between DVs for each group of the IVs, as assessed by scatterplots; there was no multicollinearity (the highest r was 0.56); there was independence of observations; DVs were continuous; and IV categorical (Fisher's exact test was performed for dichotomous DVs).

There was statistically significant difference between 1st and 2nd half of the meeting on the combined dependent variables, F(11, 809) = 21.56, p < .001; Hotelling's Trace = 0.29. partial η^2 = .23. Follow-up univariate ANOVAs showed that scores on the **quality or information and contribution** were statistically lower in the 2nd half of the meeting, as well as the scores on the certain aspects of the quality of interactions including **asking questions** and **positive reactions**. In contrast, *negative reactions* were statistically higher in the 2nd half. **In terms of the internal and external factors**, *time-workload ratio* and *clinical complexity* were statistically lower in the 2nd half, while group size was significantly higher. The remaining variables did not reach statistical significance. See Table 4 for means, standard deviations and univariate ANOVA statistics (controlling for tumor type).

In Table 5 we present correlation coefficients between the quality of DM, interactions, case-complexity, external and internal factors with the decision count across two equal temporal halves, and across the entire dataset.

For the 1st half of the meeting, there are fewer significant correlations with only the quality of contribution to discussion showing a negative association i.e. a decrease with each treatment decision made. **For the 2nd half of the meeting** however, the majority of the associations became significantly negative, with the exception of the quality of information and positive reactions which remained non-significant (although negative). On the **overall sample** the pattern remains largely the same as in the 2nd half of the meeting with the exception of the quality of information and positive reactions which turned significantly

negative. This indicates that quality of DM including information and contribution quality, as well as certain aspects of the interaction process, namely, *asking questions* and *positive reactions* decline with each subsequent treatment recommendation.

For the case-complexity, it is evident that the overall score is negatively associated with the ordinal decision counter, indicating that as the meeting moves forwards, patient-discussions become less complex. This is also the case for the individual components i.e. the *logistical and clinical complexities,* although in the 1st half of the meeting, the complexity does not seem to be related to the serial position indicating a more random dispersion of cases.

Team composition does not appear consistent during meetings. For instance, *group size* and *professional diversity* both appear positively correlated with the serial decision counter in the 1st half of the meeting, indicating that the team members and disciplinary groups are joining the meeting as patients are being discussed. In contrast, in the 2nd half of the meeting, the association is negative, indicating the opposite effect i.e. that the team members and professional groups are beginning to leave the meeting as it progresses. Moreover, as the meeting progresses increasingly fewer males and more females are in attendance; this is evident throughout, although some variation in the strength of these relationships exist in 2nd half of the meeting

Disciplinary distribution also appears to be negatively associated with the serial decision counter indicating that as the meeting progresses there is an increased inequality in the number of people present within each discipline – an effect evident in the 1st half of the meeting and somewhat weaker on the overall dataset. However, in the 2nd half, this relationship is non-significant and close to zero pointing to a more balanced disciplinary distribution.

Partial correlations

Partial correlation analysis controlling for tumour type was conducted to further explore the differences between 1st and 2nd half of the meeting.

For the 1st half of the meeting, there are fewer significant correlations with only the quality of contribution to discussion showing a negative association i.e. a decrease with each treatment decision made. **For the 2nd half of the meeting** however, the majority of the

associations became significantly negative, with the exception of the quality of information and positive reactions which remained non-significant (although negative). On the **overall sample** the pattern remains largely the same as in the 2nd half of the meeting with the exception of the quality of information and positive reactions which turned significantly negative.

For the case-complexity, it is evident that the overall score is negatively associated with the ordinal decision counter, indicating that as the meeting moves forwards, patient-discussions become less complex. This is also the case for the individual components i.e. the *logistical and clinical complexities,* although in the 1st half of the meeting, the complexity does not seem to be related to the serial position indicating a more random dispersion of cases.

Team composition does not appear consistent during meetings. For instance, *group size* and *professional diversity* both appear positively correlated with the serial decision counter in the 1st half of the meeting, indicating that the team members and disciplinary groups are joining the meeting as patients are being discussed. In contrast, in the 2nd half of the meeting, the association is negative, indicating the opposite effect i.e. that the team members and professional groups are beginning to leave the meeting as it progresses. Also, as the meeting progresses increasingly fewer males and more females are in attendance; this is evident throughout, although some variation in the strength of these relationships exist in 2nd half of the meeting.

Disciplinary distribution also appears to be negatively associated with the serial decision counter indicating that as the meeting progresses there is an increased inequality in the number of people present within each discipline – an effect evident in the 1st half of the meeting and somewhat weaker on the overall dataset. However, in the 2nd half, this relationship is non-significant and close to zero pointing to a more balanced disciplinary distribution.

References

Hair, J. F., Black, W. C., Babin, B. J., & Anderson, R. E. (2014). *Multivariate data analysis* (7th ed.). Harlow, England: Pearson.

Jaccard, J. (1998). *Interaction effects in factorial analysis of variance*. Thousand Oaks, CA: Sage Publications.

Maxwell, S. E., & Delaney, H. D. (2004). *Designing experiments and analyzing data: A model comparison perspective* (2nd ed.). New York: Psychology Press.

Soukup T. Socio-cognitive factors that affect decision-making in cancer multidisciplinary team meetings [PhD Thesis; Clinical Medicine Research]. Imperial College London. London, UK; 2017.

2. Descriptive statistics across individual items of the tools used in the study

2.1 MeDiC (case-complexity)

2.2 MDT-MODe (decision-making)

2.3 Bales’ IPA (interaction process)

2.1 Descriptive Statistics for Individual Items of the Measure of case-Discussion Complexity, MeDiC

**MeDiC** Items	**Breast team** (*n* = 241)	**Colorectal team** (*n* = 185)	**Gynaecological team** (*n* = 396)	**Overall** (*N*= 822)
	Sum	Sum	Sum	Sum
1. Malignancy	113	124	204	441
2. Invasive component	80	81	90	251
3. Residual tumour	13	9	25	47
4. Recurrence	5	10	28	43
5. Multiple cancers	21	20	27	68
6. Increased size (T3, T4)	18	51	11	80
7. Nodes affected	32	43	28	103
8. Mets (local or distant)	32	39	39	110
9. Advanced stage, progressive	28	25	49	102
10. Unusual or rare tumour type	9	11	14	34
11. Previous history of cancer	21	31	37	89
12. Previous oncological treatments	16	21	10	47
13. Significant surgical history	21	34	27	82
14. Significant physical comorbidity	20	42	52	114
15. Mental health/ cognitive comorbidity	3	8	2	13
16. Socio-economic issues	2	1	0	3
17. Lifestyle risks	0	3	4	7
18. Patient choice and family opinion	19	15	28	62
19. Diagnostic uncertainty	33	40	32	105
20. Further tests and patient assessment needed	63	75	97	235
21. Further input needed from other specialties	22	39	54	115
22. Unusual anatomy/ distribution of tumour	2	22	12	36
23. Guidelines do not account for patients situation	1	0	0	1
24. Conflict of opinions about treatment options	21	3	23	47
25. Treatment toxicity and contraindications	2	1	2	5
26. Trial eligibility	1	1	2	4
	***M* (*SD*)**	***M* (*SD*)**	***M* (*SD*)**	***M* (*SD*)**
27. Logistical complexity (frequency count)	2.52 (2.5)	4.05 (2.5)	2.26 (2.4)	2.74 (2.6)
Total clinical complexity (sum of items 1 to 26)	4 (4)	6 (4)	3 (4)	4 (4)
Total complexity (sum of clinical and logistical)	2.96 (2.7)	4.95 (2.7)	2.67 (2.5)	3.27 (2.7)

Note. All scores are frequency counts conducted per patient-discussion within each meeting. MeDiC = Measure of Discussion Complexity. Reprinted with permission from Soukup T. Socio-cognitive factors that affect decision-making in cancer multidisciplinary team meetings [PhD Thesis; Clinical Medicine Research]. Imperial College London. London, UK; 2017.

2.2 Descriptive Statistics for Individual Items of the Metric for the Observation of Decision-Making in Cancer Multidisciplinary Teams, MDT-MODe

	Breast team (*n* = 241)			Colorectal team (*n* = 185)			Gynaecological team (*n* = 396)			Overall (*N* = 822)		
**MDT-MODe Items**	*M* (*SD*)	*Mdn* (*IQR*)	Min, Max	*M* (*SD*)	*Mdn* (*IQR*)	Min, Max	*M* (*SD*)	*Mdn* (*IQR*)	Min, Max	*M* (*SD*)	*Mdn* (*IQR*)	Min, Max
1. Patient history	2.56 (3)	3 (1)	1, 4	2.64 (0.78)	3 (1)	1, 5	2.51 (0.82)	2 (1)	1, 5	2.55 (0.79)	3 (1)	1, 5
2. Radiology	2.50 (1.73)	1 (3)	1, 5	3.83 (1.78)	5 (4)	1, 5	3.67 (1.79)	5 (4)	1, 5	3.36 (1.86)	4 (4)	1, 5
3. Histopathology	3.42 (1.24)	4 (0)	1, 5	2.04 (1.48)	1 (3)	1, 5	2.44 (1.74)	1 (3)	1, 5	2.64 (1.64)	3 (3)	1, 5
4. Psychosocial	1.12 (0.48)	1 (0)	1, 4	1.16 (0.57)	1 (0)	1, 5	1.05 (0.29)	1 (0)	1, 3	1.09 (0.43)	1 (0)	1, 5
5. Comorbidities	1.15 (0.55)	1 (0)	1, 4	1.23 (0.69)	1 (0)	1, 5	1.17 (0.70)	1 (0)	1, 5	1.18 (0.66)	1 (0)	1, 5
6. Patient views	1.18 (0.75)	1 (0)	1, 5	1.11 (0.53)	1 (0)	1, 5	1.07 (0.44)	1 (0)	1, 5	1.11 (0.57)	1 (0)	1, 5
Information score (sum of 1 to 6)*	11.92 (2.65)	11 (4)	6, 23	12.01 (2.73)	12 (3)	6, 25	11.91 (2.50)	11 (2)	6, 22	11.93(2.6)	12 (2)	6, 25
7. Surgeons' input	3.57 (1.54)	4 (2)	1, 5	4.21 (1.29)	5 (4)	1, 5	3.69 (1.36)	4 (2)	1, 5	3.77 (1.42)	4 (2)	1, 5
8, Oncologists' input	2.27 (1.79)	1 (4)	1, 5	2.25 (1.77)	1 (4)	1, 5	1.51 (1.23)	1 (0)	1, 5	1.90 (1.59)	1 (2)	1, 5
9. Nurses' input	1.45 (1.18)	1 (0)	1, 5	2.46 (1.72)	1 (4)	1, 5	1.36 (1.02)	1 (0)	1, 5	1.63 (1.33)	1 (0)	1, 5
10. Radiologists' input	1.80 (1.48)	1 (0)	1, 5	2.92 (1.88)	3 (4)	1, 5	2.48 (1.74)	1 (3)	1, 5	2.38 (1.75)	1 (3)	1, 5
11. Pathologists' input	2.32 (1.73)	1 (3)	1, 5	1.74 (1.45)	1 (0)	1, 5	2.24 (1.74)	1 (3)	1, 5	2.15 (1.69)	1 (3)	1, 5
Contribution score (sum of 7 to 11)*	11.41 (4.77)	11 (6)	5, 25	13.59 (4.23)	13 (6)	5, 23	11.29 (3.79)	11 (5)	5, 25	11.84 (4.3)	12 (6)	5, 25

Note. *Information score ranges from 5 to 30. †Contribution score from 5 to 25. Individual items are scored on a range from 1 to 5 with higher scores indicating better quality. Reprinted with permission from Soukup T. Socio-cognitive factors that affect decision-making in cancer multidisciplinary team meetings [PhD Thesis; Clinical Medicine Research]. Imperial College London. London, UK; 2017.

2.3 Descriptive Statistics for Individual Items of the Bales' Interaction Process Analysis, Bales' IPA

Bales' IPA Items	Breast team (*n* = 241)			Colorectal team (*n* = 185)			Gynaecological team (*n* = 396)			Overall (*N* = 822)		
	M (*SD*)	*Mdn* (*IQR*)	Min, Max	*M* (*SD*)	*Mdn* (*IQR*)	Min, Max	*M* (*SD*)	*Mdn* (*IQR*)	Min, Max	*M* (*SD*)	*Mdn* (*IQR*)	Min, Max
1. Shows solidarity	0.56 (0.99)	0.00 (1)	0, 5	0.31 (0.77)	0.00 (0)	0, 5	0.27 (0.67)	0.00 (0)	0, 6	0.37 (0.81)	0.00 (0)	0, 6
2. Tension release	0.91 (1.45)	0.00 (1)	0, 7	0.30 (0.70)	0.00 (0)	0, 3	0.86 (1.52)	0.00 (1)	0, 9	0.75 (1.37)	0.00 (1)	0, 9
3. Agrees	3.30 (3.56)	2 (4)	0, 26	2.18 (2.31)	2 (3)	0, 12	1.37 (1.77)	1 (2)	0, 12	2.12 (2.66)	1 (3)	0, 26
Positive reactions (sum of 1 to 3)	4.77 (4.79)	4 (6)	0, 32	2.8 (2.96)	2 (3)	0, 14	2.51 (2.58)	2 (3)	0, 14	3.24 (3.58)	2 (3)	0, 32
4. Gives suggestion	3.40 (1.95)	3 (2)	1, 12	2.81 (1.59)	2 (2)	1, 11	2.72 (1.75)	2 (2)	0, 11	2.94 (1.8)	2 (2)	0, 12
5. Gives opinion	5.73 (6.79)	3 (8)	0, 38	4.40 (5.44)	2 (5)	0, 30	3.24 (4.72)	1 (5)	0, 26	4.23 (5.65)	2 (6)	0, 38
6. Gives suggestion	5.10 (4.27)	4 (5)	0, 23	7.70 (6.08)	7 (8)	0, 30	7.44 (5.31)	7 (7)	0, 28	6.82 (5.33)	6 (7)	0, 30
Gives answers (sum of 3 to 6)	14.23 (10.7)	11 (12)	2, 60	14.91 (10.7)	12 (10.5)	2, 60	13.40 (9.21)	11 (9.75)	1, 56	13.98 (10.0)	11 (11)	1, 60
7. Asks for orientation	3.84 (4.31)	2 (5)	0, 21	5.55 (4.86)	5 (5)	0, 31	4.77 (4.44)	4 (4)	0, 30	4.67 (4.54)	4 (5)	0, 31
8. Asks for opinion	1.46 (2.18)	0.00 (2)	0, 10	1.79 (2.87)	1 (3)	0, 19	0.96 (2.43)	0.00 (1)	0, 19	1.29 (2.49)	0.00 (2)	0, 19
9. Asks for suggestion	0.36 (0.71)	0.00 (1)	0, 3	0.28 (0.63)	0.00 (0)	0, 4	0.06 (0.43)	0.00 (0)	0, 7	0.20 (0.58)	0.00 (0)	0, 7
Asks questions (sum of 6 to 9)	5.66 (5.99)	4 (8)	0, 30	7.61 (7.47)	6 (7)	0, 50	5.79 (6.59)	4 (5)	0, 51	6.16 (6.67)	4 (6)	0, 51
10. Disagrees	2.29 (2.72)	1 (4)	0, 13	1.68 (2.05)	1 (3)	0, 10	0.87 (1.58)	0.00 (1)	0, 10	1.47 (2.17)	0.00 (2)	0, 13
11. Shows tension	1.02 (2.97)	0.00 (0)	0, 22	0.97 (2.66)	0.00 (0)	0, 14	0.42 (1.29)	0.00 (0)	0, 7	0.72 (2.25)	0.00 (0)	0, 22
12. Shows antagonism	0.59 (1.20)	0.00 (1)	0, 9	1.12 (1.46)	1 (2)	0, 6	0.13 (0.67)	0.00 (0)	0, 8	0.49 (1.29)	0.00 (0)	0, 9
Negative reactions (sum of 10 to 12)	3.9 (4.15)	3 (5)	0, 23	3.77 (3.39)	3 (5)	0, 15	1.41 (2.00)	0.00 (2)	0, 10	2.67 (3.32)	2 (4)	0, 23

Note. All scores are frequency counts conducted per patient-discussion within each meeting. Reprinted with permission from Soukup T. Socio-cognitive factors that affect decision-making in cancer multidisciplinary team meetings [PhD Thesis; Clinical Medicine Research]. Imperial College London. London, UK; 2017.

3. Interrater reliability across individual items of the tools used in the study

3.1 MeDiC (case-complexity)

3.2 MDT-MODe (decision-making)

3.3 Bales' IPA (interaction process)

3.1 Intraclass Correlation Coefficients (ICCs) for Individual Items of the Measure of case-Discussion Complexity, MeDiC

Reliability statistics for MeDiC have been published previously, and can be found here:

- Soukup T, Morbi MA, Lamb BW, et al. A measure of case complexity for streamlining workflow in cancer multidisciplinary tumor boards: Mixed methods development and early validation of the MeDiC tool. Cancer Med. 2020;00:1–12. https://doi.org/10.1002/cam4.3026

- Soukup T. Socio-cognitive factors that affect decision-making in cancer multidisciplinary team meetings [PhD Thesis; Clinical Medicine Research]. Imperial College London. London, UK; 2017.

3.2 Intraclass Correlation Coefficients (ICCs) for Individual Items of the Observation of Decision-Making in Cancer Multidisciplinary Teams, MDT-MODe

	MDT-MODe Items	Intraclass Correlation	
		Coefficient	95% CI
1.	Patient history	.715	.629 - .784
2.	Radiology	.869	.825 - .903
3.	Pathology	.710	.623 - .779
4.	Psychosocial information	.792	.726 - .844
5.	Comorbidity	.872	.829 - .905
6.	Patient view	.809	.877 - .932
Information score (sum of 1 to 6)		.863	.817 - .898
7.	Surgeon	.914	.884 - .936
8.	Oncologist	.896	.865 - .926
9.	Nurse	.877	.841 - .912
10.	Radiologist	.951	.934 - .964
11.	Pathologist	.933	.909 - .951
Contribution score (sum of 7 to 11)		.948	.928 - .962

Note. Number of cases rated was 158 (20%). MDT-MODe = Metric for the Observation of Decision-making in cancer multidisciplinary Teams. Reprinted with permission from Soukup T. Socio-cognitive factors that affect decision-making in cancer multidisciplinary team meetings [PhD Thesis; Clinical Medicine Research]. Imperial College London. London, UK; 2017.

3.3 Intraclass Correlation Coefficients (ICCs) for Individual Items of the Bales' Interaction Process Analysis, Bales' IPA

#	Bales' IPA ItemS	Intraclass Correlation	
		Coefficient	95% CI
1.	Shows solidarity	.953	.933 - .967
2.	Tension release	.962	.945 - .973
3.	Agrees	.978	.967 - .985
4.	Gives suggestions	.953	.933 - .968
5.	Gives opinion	.979	.970 - .985
6.	Gives orientation	.990	.985 - .993
7.	Asks orientation	.983	.975 - .988
8.	Asks opinion	.986	.979 - .990
9.	Asks suggestions	.954	.935 - .968
10.	Disagrees	.974	.968 - .982
11.	Shows tension	1.000	-
12.	Shows antagonism	.977	.968 - .984

Note. Number of cases rated was 117 (15%). Bales' IPA = Bales' Interaction Process Analysis. Reprinted with permission from Soukup T. Socio-cognitive factors that affect decision-making in cancer multidisciplinary team meetings [PhD Thesis; Clinical Medicine Research]. Imperial College London. London, UK; 2017.

4. Logistical issues encountered by the teams during their meetings

4.1 Type of logistical issues

4.2 Frequency of logistical issues

4.1 Type of logistical issues

I. Admin errors

- Side of lesion is mixed up
- Patient's DOB or name spelling is incorrect and so their radiology images or pathology results cannot be found

II. Process issues

- Insufficient details on request/ referral forms or reports from for e.g. other hospital, MDT, or GP
- One of the core members needs to leave the meeting to obtain missing information/ report
- Radiology or pathology results not ready or not yet done
- Team is not sure why is the patient on MDT list or why certain tests were performed
- There are problems with diagnostic equipment so tests were not done in time for the MDT
- There are issues with appointments and who is going to follow-up with the patient due to overbooking
- There are issues with outsourcing tests and non-standardised forms so some information or results are missing or delayed, and need to be chased up

III. Attendance

- One of the core members that is needed to make a decision is not present so decision cannot be reached at this point and case needs to be re-discussed when the member arrives. For e.g. there is no radiologist (or is running late) and so patients that need radiology input cannot be discussed which leads to them being discussed again later in the meeting (i.e. twice), or those that need oncologist input may need to be re-discussed again later if oncologist is not there, or the responsible clinician is not around and the team feels that they are not able to make a treatment plan until they arrive
- No one present has seen the patient, and so there is insufficient information to make treatment plan and the patient needs to be re-discussed the following week

IV. Issues with the meeting equipment

- Team is not able to able to connection with another site (e.g. using videoconferencing) which provides input from disciplines and specialties that are not able to be physically

present in the meetings; this means that the discussion for patients needing the input from them is delayed and will need to be repeated later in the meeting or next week

- Slides are not working and so pathology and imaging cannot be shown to the team
- Computer system is slow or not working and so patient information (for e.g., written pathology report) cannot be accessed or retrieved, and so the patient needs to be postponed for the following week

---

Reprinted with permission from Soukup T. Socio-cognitive factors that affect decision-making in cancer multidisciplinary team meetings [PhD Thesis; Clinical Medicine Research]. Imperial College London. London, UK; 2017.

4.2 Frequency of logistical issues across teams and overall dataset

Cancer Team	*N*	Number of logistical problems											
		**0**		**1**		**2**		**3**		**4**		**Total**	
		count	%	count	%	count	%	Count	%	count	%	count	%
Breast	241	157	32	67	28	13	2	4	0.7	-	-	84	29
Gynaecological	396	262	8	111	45	17	4	6	0.9	-	-	**134**	48
Colorectal	185	66	19	85	28	29	2	7	0.5	1	0.5	121	23
Overall	822	**472**	59	**261**	32	59	7	16	2.1	1	0.1	**339**	41

Note. % is a percentage of the total count. 42% of all patient-discussions encountered a logistical problem with the highest frequency appearing in the gynaecological and colorectal teams. One logistical issue per discussion appears most common throughout, with two being somewhat less frequent, and three and four the least common. Reprinted with permission from Soukup T. Socio-cognitive factors that affect decision-making in cancer multidisciplinary team meetings [PhD Thesis; Clinical Medicine Research]. Imperial College London. London, UK; 2017.

5. Copy of the tools used in the study

5.1 MeDiC (case-complexity)

5.2 MDT-MODe (decision-making)

5.3 Bales' IPA (interaction process)

5.1 Metric for the observation of decision-making in cancer multidisciplinary team meetings, MDT-MODe

MTB-MODe Metric for the Observation of Decision making II

Whipps Cross University Hospital NHS Trust — Building Futures Together | Imperial College London | CPSSQ

			Information						Contribution							OUTCOME	
#	Site	Point	Hx	X-ray	Path	Psy/soc	Co-morbid	Patient view	Chair	Surg	Phys	Oncolo	Nurse	Radiolo	Histopath	Y/D/N	Free text
1																	
2																	

History	5	Fluent, comprehensive case history.	Psycho-social	5	Comprehensive first-hand knowledge of patients' personal circumstances, social and psychological issues.
	3	Partial case history.		3	Vague first-hand knowledge, or good second-hand knowledge of personal circumstances, social and psychological issues.
	1	No patient case history.		1	No knowledge of personal circumstances, social and psychological issues
x-ray	5	Radiological images.	Co-morbidity	5	Comprehensive first-hand knowledge of patients' past medical history and performance status.
	3	Radiological information from a report/ account.		3	Vague first-hand knowledge, or good second-hand knowledge of past medical history or performance status.
	1	No provision of radiological information.		1	No knowledge of past medical history or performance status.
Pathology	5	Histopathological information explained with slides/pictures.	Patient's views	5	Comprehensive first-hand knowledge of patients' wishes or opinions regarding treatment.
	3	Histopathological information from a report/account.		3	Vague first-hand knowledge, or good second-hand knowledge of patient's wishes or opinions regarding treatment.
	1	No provision of Histopathological information.		1	No knowledge of patient's wishes or opinions regarding treatment.
Chair	5	Good leadership enhanced team discussion and decision making.	Members	5	Clear contribution of speciality.
	3	Leadership neither enhanced nor impeded team discussion and decision making.		3	Contribution inarticulate or vague.
	1	Poor/inadequate leadership impeded team discussion and decision making.		1	No contribution.
Point	Pre Rx	Pre-treatment.	Decision	Y	Clear decision about treatment(s) to be offered.
	Post Rx	Post treatment.		D	Decision to defer to next MDT.
	R	Recurrence/ surveillance.		N	No decision/decision unclear.

Note. Copyright 2014 @ Lamb Sevdalis Green. **Developed** by: Lamb BW, Wong HWL, Vincent C, Green JSA, Sevdalis N. Teamwork and team performance in multidisciplinary cancer teams: Development of an observational assessment tool. BMJ Qual Saf 2011;20:849-856. **Further validated** by: Soukup T, Lamb BW, Sarkar S, Arora S, Shah S, Darzi A, Green JSA, Sevdalis N. Predictors of treatment decision in multidisciplinary oncology meetings: A quantitative observational study. Ann Surg Oncol 2016;23(13):4410-4417. Soukup T, Petrides KV, Lamb BW, Sarkar S, Arora S, Shah S, Darzi A, Green JSA, Sevdalis N. The anatomy of clinical decision-making in multidisciplinary cancer meetings: A cross-sectional observational study of teams in a natural context. Medicine 2016;95;e3885.

5.2 Bales' interaction process analysis, Bales' IPA

Imperial College London

National Institute for Health Research

Bales Interaction Process Analysis for Cancer MDMs

Case No.	Positive reactions (emotion)			Attempted answers (task)			Questions (task)			Negative reactions (emotion)		
	(1) Shows solidarity / cooperation / gives help / raises others status/ friendly	(2) Tension release/ PFR /dramatizes /self-revealing /jokes, laughs / shows satisfaction	(3) Agrees / passive acceptance / understands/ concurs /complies/ unassertive	(4) Gives suggestions/ direction/instruction/ solution/way to achieve goal/ where to start/ management	(5) Gives opinion/ evaluation/ interpretation/ decision-making / reasoning /inference	(6) Gives orientation / factual information / repeats / clarifies / confirms	(7) Asks for orientation /factual information / repetition / confirmation / clarification	(8) Asks for opinion/ interpretation / evaluation / analysis / inference	(9) Asks for suggestion / direction / instruction/ solution/ possible ways of action/goal	(10) Disagrees/ hesitant/ critical/ passive rejection/ unacknowledging / doing something other than task – e.g. whispering	(11) Shows tension/ fear of provoking opposition/ nervous, frustr./ concerned/ self-critical	(12) Shows antagonism / status deflating/ self-assertive/ interrupts/ ignores direction / autocratic
1												
2												
3												
4												
5												
6												
7												
8												
9												
10												

Note. Note originator & target. C=chair, R=radiologist, P=pathologist, N=nurse, O=oncologist, S=surgeon, MDTC=coordinator, T=team, Y=self (activity directed toward self), X=non-member/ absent person. E.g. T-C = all team members to chair.

Note. **Developed** by**:** Bales RF. Personality and Interpersonal Behaviour. New York, Rhinehart and Winston; 1970. **Adapted for cancer MDTs** by: Soukup T. Socio-cognitive factors that affect decision-making in cancer multidisciplinary team meetings [PhD Thesis; Clinical Medicine Research]. Imperial College London. London, UK; 2017.

5.3 Measure of case-discussion complexity for cancer team meetings, MeDiC

#	MeDiC items
**Pathology**	
1	Malignancy
2	Invasive component
3	Residual tumour
4	Recurrence
5	...
6	...
7	...
8	...
9	...
10	...
**Patient factors**	
11	...
12	...
13	Significant surgical history
14	Significant physical comorbidity
15	Mental health and cognitive comorbidity
16	Socio-economic issues
17	...
18	...
**Treatment factors**	
19	Diagnostic uncertainty / inconclusiveness
20	Further tests and patient assessment needed
21	Further input needed from other specialties
22	Unusual anatomy/ distribution of tumour
23	...
24	...
25	...
26	Trial eligibility
**Logistical complexity** (frequency counts)	
Total clinical complexity (sum of 1 to 26)	
Total complexity (sum of 1 to 27)	

Note. Copyright 2017 @ Soukup Sevdalis Green. **Developed by:** Soukup T. Socio-cognitive factors that affect decision-making in cancer multidisciplinary team meetings [PhD Thesis; Clinical Medicine Research]. Imperial College London. London, UK; 2017. Soukup T, Morbi MA, Lamb BW, Gandamihardja T, Hogben K, Noyes K, Skolarus TA, Darzi A, Sevdalis N, Green JSA. A measure of case complexity for streamlining workflow in cancer multidisciplinary tumor boards: Mixed methods development and early validation of the MeDiC tool. Cancer Med 2020;00:1–12.

6. STROBE statement—checklist of items that should be included in reports of observational studies

	Item No	Recommendation	Page No
**Title and abstract**	1	(*a*) Indicate the study's design with a commonly used term in the title or the abstract	1
		(*b*) Provide in the abstract an informative and balanced summary of what was done and what was found	2
**Introduction**			
Background/rationale	2	Explain the scientific background and rationale for the investigation being reported	3-4
Objectives	3	State specific objectives, including any prespecified hypotheses	5
**Methods**			
Study design	4	Present key elements of study design early in the paper	5
Setting	5	Describe the setting, locations, and relevant dates, including periods of recruitment, exposure, follow-up, and data collection	5
Participants	6	(*a*) *Cohort study*—Give the eligibility criteria, and the sources and methods of selection of participants. Describe methods of follow-up<br>*Case-control study*—Give the eligibility criteria, and the sources and methods of case ascertainment and control selection. Give the rationale for the choice of cases and controls<br>*Cross-sectional study*—Give the eligibility criteria, and the sources and methods of selection of participants	5-7
		(*b*) *Cohort study*—For matched studies, give matching criteria and number of exposed and unexposed<br>*Case-control study*—For matched studies, give matching criteria and the number of controls per case	
Variables	7	Clearly define all outcomes, exposures, predictors, potential confounders, and effect modifiers. Give diagnostic criteria, if applicable	7
Data sources/ measurement	8*	For each variable of interest, give sources of data and details of methods of assessment (measurement). Describe comparability of assessment methods if there is more than one group	7
Bias	9	Describe any efforts to address potential sources of bias	7,21
Study size	10	Explain how the study size was arrived at	7

Quantitative variables	11	Explain how quantitative variables were handled in the analyses. If applicable, describe which groupings were chosen and why	7
Statistical methods	12	(*a*) Describe all statistical methods, including those used to control for confounding	7
		(*b*) Describe any methods used to examine subgroups and interactions	
		(*c*) Explain how missing data were addressed	
		(*d*) *Cohort study*—If applicable, explain how loss to follow-up was addressed<br>*Case-control study*—If applicable, explain how matching of cases and controls was addressed<br>*Cross-sectional study*—If applicable, describe analytical methods taking account of sampling strategy	
		(*e*) Describe any sensitivity analyses	
**Results**			
Participants	13*	(a) Report numbers of individuals at each stage of study—eg numbers potentially eligible, examined for eligibility, confirmed eligible, included in the study, completing follow-up, and analysed	
		(b) Give reasons for non-participation at each stage	n/a
		(c) Consider use of a flow diagram	n/a
Descriptive data	14*	(a) Give characteristics of study participants (eg demographic, clinical, social) and information on exposures and potential confounders	6
		(b) Indicate number of participants with missing data for each variable of interest	n/a
		(c) *Cohort study*—Summarise follow-up time (eg, average and total amount)	n/a
Outcome data	15*	*Cohort study*—Report numbers of outcome events or summary measures over time	*n/a*
		Case-control study—Report numbers in each exposure category, or summary measures of exposure	*n/a*
		Cross-sectional study—Report numbers of outcome events or summary measures	*9-18*
Main results	16	(*a*) Give unadjusted estimates and, if applicable, confounder-adjusted estimates and their precision (eg, 95% confidence interval). Make clear which confounders were adjusted for and why they were included	n/a
		(*b*) Report category boundaries when continuous variables were categorized	n/a

		(*c*) If relevant, consider translating estimates of relative risk into absolute risk for a meaningful time period	n/a
Other analyses	17	Report other analyses done—eg analyses of subgroups and interactions, and sensitivity analyses	n/a
**Discussion**			
Key results	18	Summarise key results with reference to study objectives	19
Limitations	19	Discuss limitations of the study, taking into account sources of potential bias or imprecision. Discuss both direction and magnitude of any potential bias	21
Interpretation	20	Give a cautious overall interpretation of results considering objectives, limitations, multiplicity of analyses, results from similar studies, and other relevant evidence	19-21
Generalisability	21	Discuss the generalisability (external validity) of the study results	21
**Other information**			
Funding	22	Give the source of funding and the role of the funders for the present study and, if applicable, for the original study on which the present article is based	23

*Give information separately for cases and controls in case-control studies and, if applicable, for exposed and unexposed groups in cohort and cross-sectional studies.

Note. An Explanation and Elaboration article discusses each checklist item and gives methodological background and published examples of transparent reporting. The STROBE checklist is best used in conjunction with this article (freely available on the Web sites of PLoS Medicine at http://www.plosmedicine.org/, Annals of Internal Medicine at http://www.annals.org/, and Epidemiology at http://www.epidem.com/). Information on the STROBE Initiative is available at www.strobe-statement.org.
